# Transfer Learning-Based Interpretable Soil Lead Prediction in the Gejiu Mining Area, Yunnan

**DOI:** 10.3390/s25134209

**Published:** 2025-07-05

**Authors:** Ping He, Xianfeng Cheng, Xingping Wen, Yan Yi, Zailin Chen, Yu Chen

**Affiliations:** 1 Faculty of Land Resources Engineering, Kunming University of Science and Technology, Kunming 650093, China; heping@kmu.edu.cn; 2 School of Fine Art and Design, Kunming University, Kunming 650214, China; yiyan@kmu.edu.cn; 3 International Research Center of Big Data for Sustainable Development Goals, Beijing 100094, China; 4 School of Earth and Environmental Sciences, Yunnan Land and Resources Vocational College, Kunming 652501, China; chengxianfeng2020@163.com (X.C.); 201310362@yngtxy.edu.cn (Z.C.); 5 Engineering Center of Yunnan Education Department for Health Geological Survey & Evaluation, Kunming 650218, China; 6 Key Laboratory of Digital Earth Science, Aerospace Information Research Institute, Chinese Academy of Sciences, Beijing 100094, China

**Keywords:** soil lead (Pb), transfer learning, SHAP analysis, small sample prediction

## Abstract

Accurate prediction of soil lead (Pb) content in small sample scenarios is often limited by data scarcity and variability in soil properties, with traditional spectral modeling methods yielding suboptimal precision. To address this, we propose a transfer learning-based framework integrated with SHAP analysis for predicting soil Pb content in the Gejiu mining area, Yunnan. Using pH data from the European LUCAS soil database as the source domain, spectral features were extracted via a 1D-ResNet model and transferred to the target domain (130 soil samples from Gejiu) for Pb prediction. SHAP analysis was applied to clarify the role of spectral characteristics in cross-component transfer learning, uncovering shared and adaptive features between pH and Pb predictions. The transfer learning model (ResNet-pH-Pb) significantly outperformed direct modeling methods (PLS-Pb, SVM-Pb, and ResNet-Pb), with an R^2^ of 0.77, demonstrating superior accuracy. SHAP analysis showed that the model retained key pH-related wavelengths (550–750 nm and 1600–1700 nm) while optimizing Pb-related wavelengths (e.g., 919 nm and 959 nm). This study offers a novel approach for soil heavy metal prediction under small sample constraints and provides a theoretical basis for understanding spectral prediction mechanisms through interpretability analysis.

## 1. Introduction

Lead (Pb), a profoundly toxic, bioaccumulative, and environmentally persistent heavy metal, presents substantial risks to ecosystems and human well-being [1,2]. Due to prolonged mining and smelting activities, Pb content in mining area soils is typically high, and the pollution exhibits significant spatial heterogeneity [3,4]. The Gejiu mining area in Yunnan, one of China’s major non-ferrous metal mining regions, suffers from particularly severe Pb pollution due to historical mining activities [5]. Therefore, accurately and rapidly assessing Pb content in the soils of mining areas and identifying the key factors influencing it is of great importance for pollution assessment and environmental management.

Visible–near infrared (Vis-NIR) spectroscopy provides non-destructive, swift, and economically efficient benefits for estimating soil Pb levels [6]. Researchers typically enhance Pb spectral signal by selecting specific bands and combine this with machine learning methods such as partial least squares (PLS) and support vector machines (SVM) to build predictive models [7,8]. However, Pb lacks direct spectral absorption features, making its signal susceptible to soil matrix interference, which limits model accuracy [9]. Deep learning methods, such as 1D residual neural networks (1D-ResNet), show great potential by extracting complex spectral features through cross-layer connections [10,11]. Yet, their reliance on large sample sizes conflicts with the limited data available in mining areas [12].

Transfer learning presents a novel approach by harnessing knowledge from a related task to boost prediction accuracy despite limited target domain data [13]. Existing studies have shown that models trained on the LUCAS dataset for predicting organic carbon and pH have achieved high accuracy in cross-region transfers [14,15,16]. Notably, current research has mainly focused on transfer within the same attribute (e.g., pH → pH), while how to use soil physicochemical properties closely related to Pb to assist Pb prediction remains an unexplored issue. Soil pH is a key factor influencing the distribution and mobility of Pb [17,18]. Variations in pH substantially influence the solubility and adsorption capacity of Pb within soil [19]. Compared to Pb, pH is more easily predicted accurately from spectral data, as it strongly correlates with spectrally active soil components such as clay minerals, organic matter, and iron oxides, which show Vis-NIR absorption features [20,21]. These components also influence Pb behavior, allowing pH to indirectly inform Pb modeling [19]. This makes pH an ideal intermediary variable, as large-scale pH data from the LUCAS dataset can be used to train a source domain model and transfer this knowledge to the small sample Pb prediction task, improving the accuracy and stability of Pb predictions.

Although transfer learning can enhance prediction performance, the lack of model interpretability limits its practical application. SHAP value helps address the “black box” problem by quantifying feature contributions [22]. While SHAP has shown interpretive potential in fields such as spectral analysis and environmental monitoring [23,24,25], its application in transfer learning models, particularly in analyzing the reuse of features across components, has not been fully explored.

Therefore, this study innovatively proposes a soil Pb prediction framework that integrates transfer learning with SHAP analysis. Its core contributions include the following: (1) Building a model with the extensive LUCAS dataset for pH and using transfer learning to improve Pb predictions in the small-sample Gejiu mining area. (2) Using SHAP values for the first time to explain how spectral features contribute to Pb prediction in this transfer learning framework, offering a theoretical foundation for heavy metal spectral prediction.

## 2. Data and Methods

### 2.1. Data Sources

This study utilizes two datasets—source domain and target domain data—to predict soil lead (Pb) content via cross-component transfer learning.

The source domain data is derived from the European LUCAS Soil Database, collected by the European Commission, comprising 19,036 surface soil samples (0–20 cm) from multiple European countries [26]. Physicochemical and spectral properties were measured using standardized protocols to ensure data consistency [27]. Spectral measurements were conducted using a FOSS XDS Rapid Content Analyzer (FOSS NIRSystems Inc., Hilleroed, Denmark; 400–2500 nm, 0.5 nm resolution) [28], downsampled to 400–2499 nm (1 nm interval, 2100 wavelength points) to align with the target domain. Given the potential correlation between pH and soil Pb chemical behaviors (such as adsorption and desorption), pH was selected as the source domain task to provide a knowledge foundation for cross-component transfer learning.

**Figure 1:**
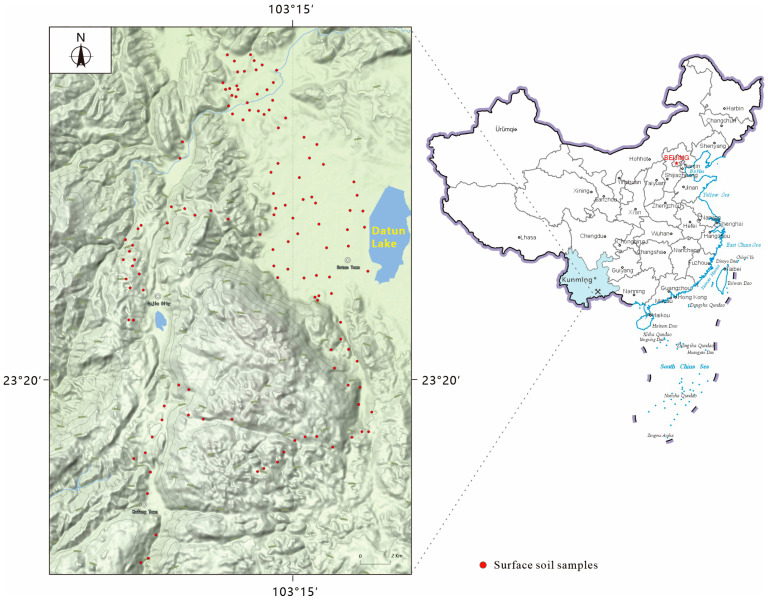
Distribution of 130 soil samples in the target domain (Gejiu mining area, Yunnan, China).

The target domain data was collected by the research team through field sampling in the Gejiu mining area, Yunnan, China, a region characterized by complex terrain and rich mineral resources, particularly tin mines [29]. Prolonged mining operations have resulted in substantial Pb pollution [30]. From March to April 2024, in the dry season when exposed soil was common, 130 surface soil samples (0–20 cm) were gathered. Sampling was carried out using a grid method: in the northern smelting area, a 1000 m × 1000 m grid was used, with denser sampling (500 m × 500 m) around the smelting plants. In the southern mining region, samples were gathered at 1000 m intervals along the road. The overall distribution of sampling points across the study area is illustrated in Figure 1. Soil samples were dried in air, had their impurities removed, ground, and sieved through a 100-mesh screen, then split into two portions: one for spectral measurement and one for Pb content analysis. Spectral measurements were obtained with an ASD FieldSpec 3 spectrometer (Analytical Spectral Devices Inc., Boulder, CO, USA; 350–2500 nm, 1 nm resolution), with five measurements averaged per sample. To reduce noise and align with the source domain, the 350–399 nm range was excluded, retaining 400–2499 nm. Pb concentrations were determined using inductively coupled plasma optical emission spectrometry (ICP-OES) after mixed-acid digestion with HNO_3_, HCl, HF, and HClO_4_ in a PTFE crucible. The residues were dissolved in 1:1 HCl and diluted to 10 mL, following established soil analysis protocols The Pb concentration data used in this study are publicly available at Zenodo: https://zenodo.org/records/15742450, accessed on 25 June 2025.

### 2.2. Methods

#### 2.2.1. ResNet Model Architecture

ResNet optimizes deep network performance through a unique residual learning mechanism, effectively addressing the degradation problem that arises with increasing network depth in traditional architectures [10]. The core design relies on residual blocks, where skip connections merge input and convolutional features, enhancing both training efficiency and model expressiveness.

**Figure 2:**
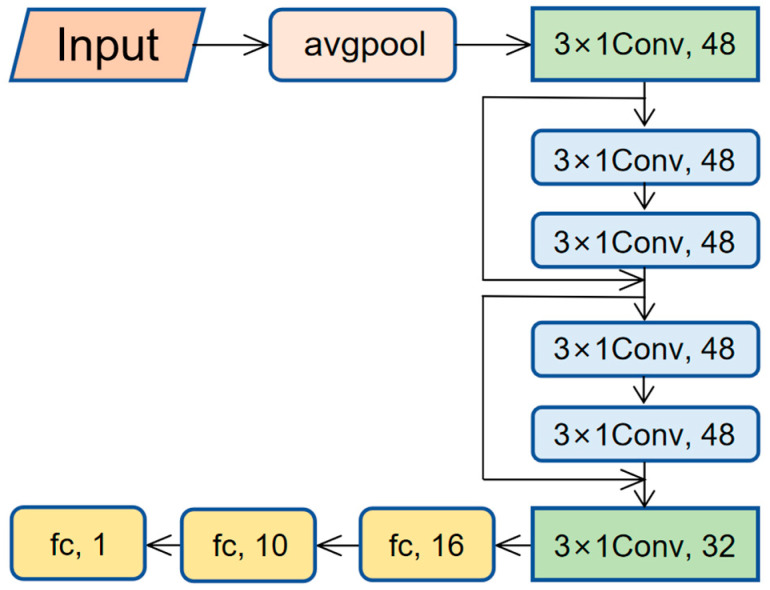
Architecture of the 1D-ResNet model used for predicting soil pH on the LUCAS dataset and applying transfer learning for Pb estimation in the Gejiu dataset.

This research developed a 1D-ResNet model utilizing spectral data characteristics. The model’s input consists of log-transformed (logR) spectral reflectance data, with a dimensionality of 2100. To improve computational efficiency and extract key features, the first layer applies average pooling with a window size of 10, reducing the dimension to 210. The data then passes through a 1D convolutional layer (48 filters, kernel size 3, stride 1, Leaky ReLU activation function with α = 0.01) for feature extraction, followed by batch normalization to optimize data distribution. The data subsequently undergoes max pooling with a window size of 2, followed by two residual blocks (with ReLU activation and skip connections). Afterward, it advances through a 1D convolutional layer (32 filters), a flattening layer, and two dense layers (16 and 10 nodes, using Leaky ReLU activation). The output layer consists of a single-node dense layer with ReLU activation. All convolutional and fully connected layers use L2 regularization (λ = 0.0004). The specific network architecture is shown in Figure 2 and Table 1.

The model was constructed using Python 3.9 and TensorFlow 2.0, employing the Adam optimizer (learning rate = 0.001) for training and utilizing mean squared error (MSE) as the loss metric. Training runs for 2000 iterations, with early stopping enabled (patience = 120), meaning training halts if no improvement is observed after 120 epochs.

#### 2.2.2. Transfer Learning Process

Transfer learning enhances target domain prediction by using rich source domain data, making it ideal for cross-component predictions such as from pH to Pb. This study employs a 1D-ResNet model with a fine-tuning strategy to transfer spectral features from the LUCAS dataset (source domain, 19,036 samples) to the Gejiu mining area (target domain, 130 samples) for improved soil Pb content prediction.

First, the source domain model, ResNet-pH, is trained on the LUCAS dataset to predict pH values, using 14,277 samples (75%) for training and 4759 samples (25%) for testing. Next, a baseline model, ResNet-Pb, is independently trained on the Gejiu dataset to directly predict Pb content, using 98 samples (75%) for training and 32 samples (25%) for testing, without relying on source domain information. For transfer learning, pre-trained weights from ResNet-pH are loaded into the target domain model. Convolutional layers and residual blocks are frozen to retain general spectral features learned from the source task (pH prediction), which often reflect fundamental soil properties such as absorption patterns of iron oxides near 550 nm. In contrast, the max-pooling layer and fully connected layers are fine-tuned using the target domain (Pb) data to capture component-specific variations. This selective updating strategy allows the model to preserve transferable low-level spectral representations while adapting to the distinct characteristics of Pb, thereby improving prediction accuracy in data-scarce conditions. The resulting transfer learning model is denoted as ResNet-pH-Pb. The model configurations are detailed in Table 2.

Both datasets are randomly split into 75% training and 25% testing sets, and this process is repeated over 10 rounds to ensure robust evaluation. The final evaluation of the model is based on the average of the evaluation metrics from the 10 test rounds. Model performance is assessed using metrics detailed in Section 2.2.4.

#### 2.2.3. Interpretability Analysis

SHAP values, based on game theory, quantify feature impacts on predictions, enhancing model interpretability [31]. This research utilizes the GradientExplainer module from the SHAP library to calculate SHAP values and assess feature importance for ResNet-based models.

For the source domain model, ResNet-pH, the LUCAS dataset’s large size (19,036 samples) leads to significant computational demands for SHAP analysis due to its need to evaluate feature interactions. We selected 1000 samples from the training set as background data to provide a baseline for estimating the model’s expected output, enabling SHAP to quantify each feature’s contribution relative to this baseline. This subset size ensures computational efficiency while capturing sufficient spectral information. From the test set, 1000 samples are selected to compute SHAP values, with their mean absolute values used to assess each wavelength’s contribution to pH prediction.

For the transfer learning model, ResNet-pH-Pb, all 130 samples from the Gejiu mining area dataset are used as background data to compute SHAP values for the test set, revealing the impact of transfer learning on Pb content prediction.

By comparing the SHAP values of ResNet-pH and ResNet-pH-Pb, we analyze differences in wavelength contributions between pH and Pb prediction tasks, elucidating how transfer learning adjusts shared and task-specific wavelengths to enhance model predictions.

#### 2.2.4. Comparison Experiments and Evaluation Metrics

To validate the effectiveness of cross-component transfer learning for soil Pb prediction in the target domain, this study trains PLS and SVM models, implemented in Python 3.9 using scikit learn, on the target domain dataset, labeled as PLS-Pb and SVM-Pb, and compares their performance with the transfer learning model ResNet-pH-Pb.

PLS extracts latent variables to model relationships between predictor and response variables [32]. It excels in handling linear relationships with low computational cost, suitable for small datasets, but may fail to capture nonlinear patterns in complex data [7,33,34]. Studies like Chen et al. (2022) reported PLS based soil Pb prediction with R^2^ = 0.59, and Arif et al. (2022) achieved R^2^ = 0.66 in urban greenbelt zones [7,34]. In our study, the PLS-Pb model was optimized using 5-fold cross validation with GridSearchCV, searching over 1 to 50 components to select 11 components for optimal performance.

SVM maps data into a higher-dimensional space using a kernel function to model complex relationships [35]. It effectively captures nonlinear relationships with flexible kernel choices, but requires careful parameter tuning and can be computationally demanding for larger datasets [1]. Chen et al. (2022) reported SVM based Pb prediction with R^2^ = 0.55 [7]. In our study, the SVM-Pb model employed a radial basis function kernel (kernel = ‘rbf’), with Bayesian optimization used to determine optimal hyperparameters, yielding C = 0.26 (regularization) and gamma = ‘scale’ (scikit-learn default setting).

Model performance is assessed using the coefficient of determination (R^2^), root mean square error (RMSE), and residual prediction deviation (RPD). R^2^ indicates the extent of variance in the data accounted for by the model, with values nearer to 1 signifying improved model fit. RMSE quantifies the average discrepancy between predicted and actual values, with lower values reflecting greater prediction precision. RPD evaluates the ratio of standard deviation to residuals to gauge prediction reliability, with RPD < 1.4 indicating inadequate prediction, 1.4 ≤ RPD < 2.0 suggesting acceptable performance, and RPD ≥ 2.0 representing superior prediction [36].

## 3. Results

### 3.1. Statistical Characterization of Soil Pb Concentrations

This study analyzed soil Pb concentrations from the Gejiu mining region and pH levels from the LUCAS dataset, with summary statistics presented in Table 3.

For LUCAS, pH values ranged from 3.21 to 10.08, with a mean of 6.02 and a median of 6.21. The skewness of −0.07 and kurtosis of −1.24 suggest a near-normal distribution with minimal asymmetry. The SD of 1.35 and CV of 0.22 indicate low variability, reflecting relatively uniform soil pH.

For Gejiu, Pb levels varied from 34.6 mg/kg to 9720 mg/kg, highlighting considerable differences in Pb concentrations across sampling locations. The average Pb level was 974.06 mg/kg, with a median of 232 mg/kg. The skewness of 3.12 and kurtosis of 9.1 suggest a markedly positively skewed distribution, indicating that most locations exhibited relatively low Pb levels, while a few showed exceptionally high concentrations. Furthermore, the standard deviation (SD) was 1969.17 mg/kg, and the coefficient of variation (CV) of 2.02 indicates substantial variability, pointing to significant spatial heterogeneity. This may be attributed to localized contamination hotspots resulting from mining activities. To mitigate the effects of skewness and variability, a logarithmic transformation was applied to the Pb levels.

### 3.2. Source Domain Modeling

**Figure 3:**
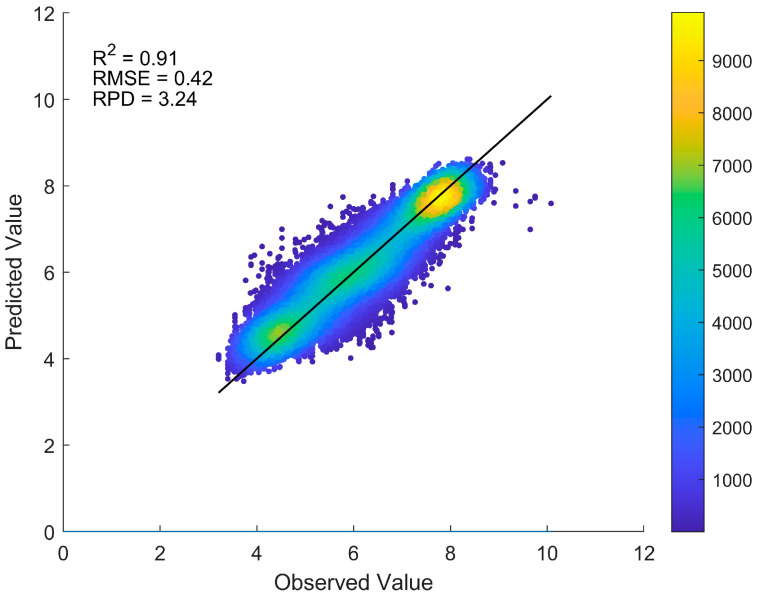
Predicted vs. observed pH for ResNet-pH model based on 10 random sampling tests. The average evaluation results of 10 test rounds are displayed in the upper-left section of the figure.

The scatter plot depicting the results of 10 rounds of random sampling tests for the source domain model (ResNet-pH) is presented in Figure 3. The model’s average R^2^ is 0.91, RMSE is 0.42, and RPD is 3.24, indicating strong predictive capability. This high-precision source domain model effectively captured the spectral features of pH values, providing a reliable foundation for subsequent target domain Pb prediction and wavelength contribution analysis.

### 3.3. Direct Modeling in the Target Domain

**Figure 4:**
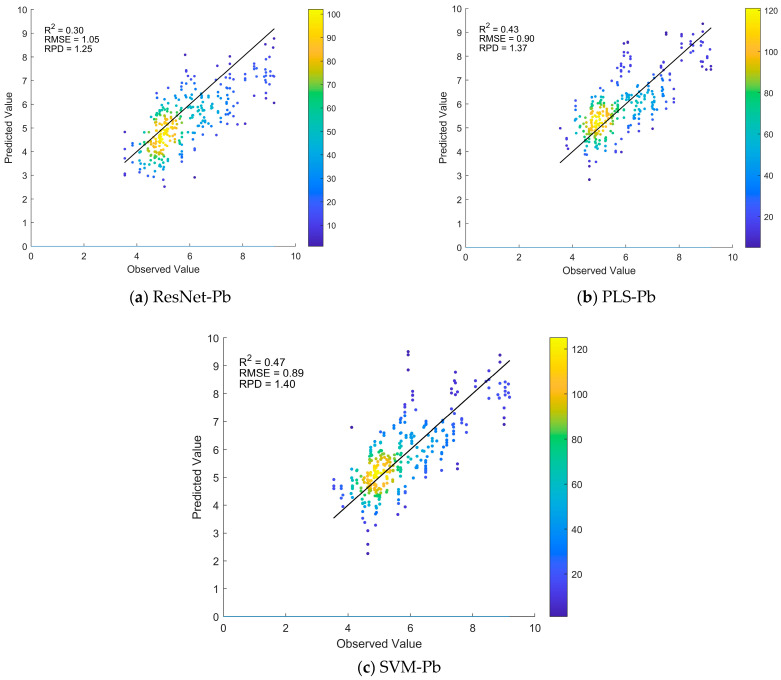
Predicted vs. observed Pb values for ResNet, PLS, and SVM models based on 10 random sampling tests in the target domain. The average evaluation metrics are shown in the upper-left corner.

For the target domain Gejiu dataset, ResNet, PLS, and SVM models were directly trained to predict Pb levels, with 10 rounds of random sampling evaluations performed. The findings are displayed in Figure 4. From the model performance, SVM-Pb achieved the best prediction results (R^2^ = 0.47, RMSE = 0.89, RPD = 1.40), followed by PLS-Pb (R^2^ = 0.43, RMSE = 0.90, RPD = 1.37), and ResNet-Pb had the worst performance (R^2^ = 0.30, RMSE = 1.05, RPD = 1.25). Compared to SVM-Pb and PLS-Pb, the R^2^ of ResNet-Pb decreased by 0.17 and 0.13, respectively. This indicates that for small sample target domains, deep learning models do not perform as well as traditional methods. Moreover, the R^2^ of all models is below 0.5, suggesting that direct modeling has limited applicability in small sample target domains.

### 3.4. Performance of the Transfer Learning Model

**Figure 5:**
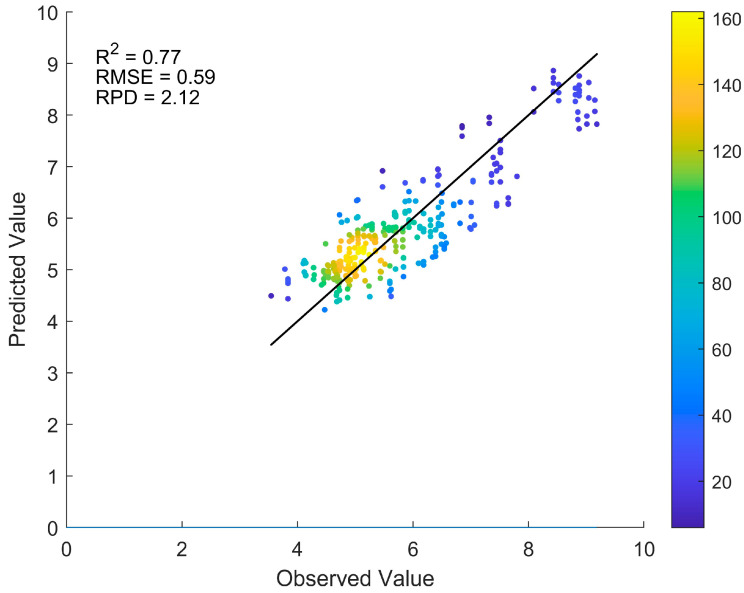
Predicted versus observed Pb values from the transfer learning model (ResNet-pH-Pb) based on 10 random sampling tests. The average evaluation metrics across tests are shown in the upper-left corner.

**Figure 6:**
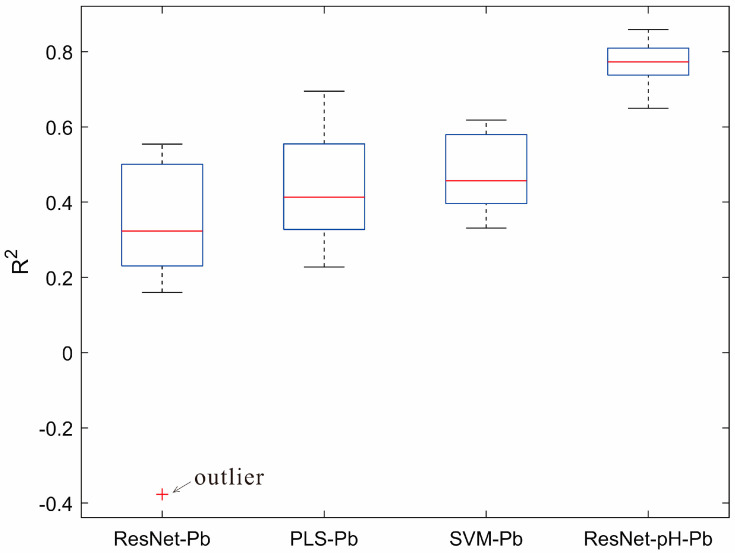
Boxplot comparison of R^2^ values for Pb prediction among the ResNet-pH-Pb, PLS-Pb, SVM-Pb, and ResNet-Pb models.

The results of 10 rounds of random sampling tests for the transfer learning model ResNet-pH-Pb are shown in Figure 5, yielding R^2^ = 0.77, RMSE = 0.59, and RPD = 2.12, significantly outperforming the direct modeling methods. The R^2^ box plot in Figure 6 shows that the average R^2^ of ResNet-pH-Pb is 0.47, 0.34, and 0.30 higher than those of ResNet-Pb, PLS-Pb, and SVM-Pb, respectively. This indicates that transfer learning not only improves Pb content prediction accuracy but also significantly enhances model stability and generalization.

### 3.5. Wavelength Contribution in ResNet Modeling

**Figure 7:**
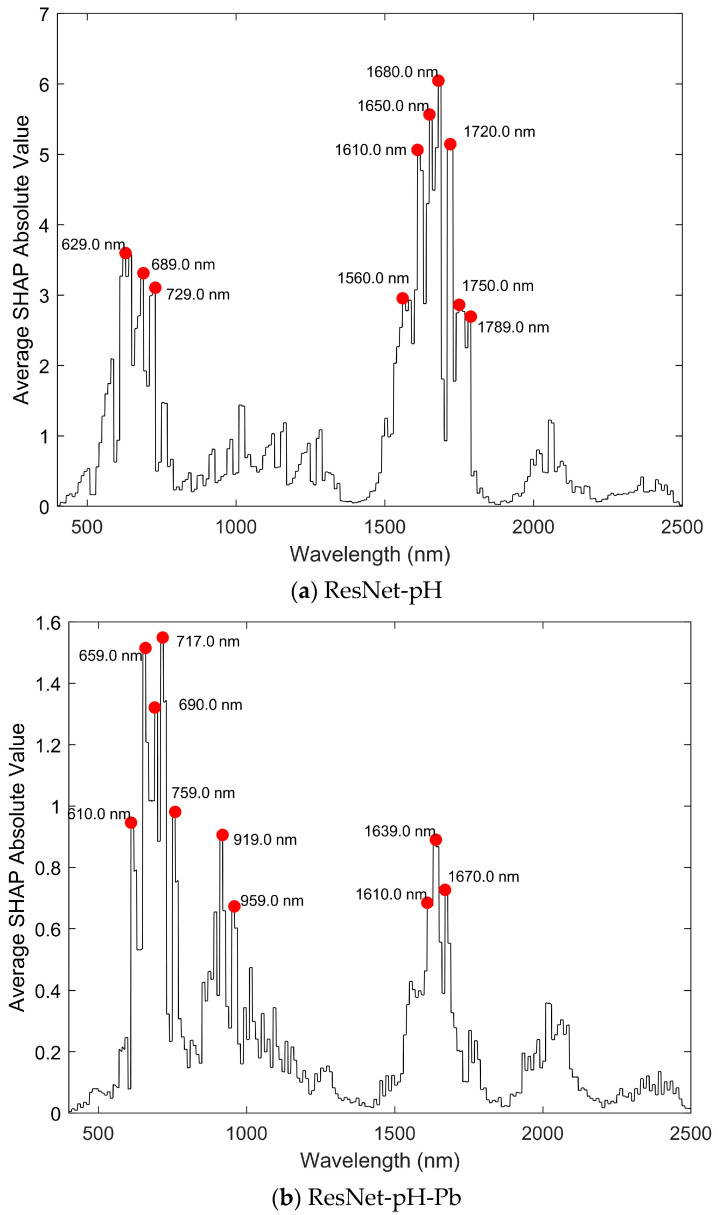
Contribution of different wavelengths to model predictions, highlighting key spectral features extracted by ResNet-pH and ResNet-pH-Pb for pH and Pb estimation.

To explore the contribution of different wavelengths in ResNet modeling, SHAP values were used to interpret the source domain model ResNet-pH and the transfer learning model ResNet-pH-Pb, as shown in Figure 7.

In the ResNet-pH model (Figure 7a), the wavelengths with significant contributions to pH prediction are mainly concentrated at 629 nm, 689 nm, 729 nm, 1560 nm, 1650 nm, 1680 nm, 1750 nm, 1789 nm, and other positions, suggesting that these wavelengths may be closely related to the spectral features of soil pH.

In contrast, in the ResNet-pH-Pb model (Figure 7b), the wavelength contributions have shifted. New key wavelengths have emerged, including 610 nm, 659 nm, 717 nm, and 759 nm in the visible range, and 919 nm, 959 nm, 1639 nm, and 1670 nm in the near-infrared range. Meanwhile, some key wavelengths from the source domain model, like 689 nm and 1610 nm, are still retained. This indicates that in the Pb prediction task, the model not only inherited part of the spectral information from the pH modeling but also focused on new wavelength regions related to Pb, especially in the near-infrared range (e.g., 919 nm and 959 nm).

Overall, the transfer learning model ResNet-pH-Pb has undergone significant adjustments in its wavelength contributions. This indicates that while the spectral behavior of soil Pb is partially related to pH, they are not identical. With the introduction of transfer learning, the model is able to more effectively focus on the important wavelengths needed for Pb prediction, enhancing the reliability of Pb content prediction.

## 4. Discussion

### 4.1. Improvement in Prediction Performance for Small Sample Target Domains Using Transfer Learning

In soil spectroscopy, the accuracy of Pb prediction is often limited by small sample sizes and high heterogeneity. In this study, direct modeling was conducted on a target domain with 130 samples from the Gejiu region. The findings indicate that the R^2^ values for SVM-Pb, PLS-Pb, and ResNet-Pb are 0.47, 0.43, and 0.30, respectively. This indicates that both traditional modeling methods and deep learning approaches struggle to achieve satisfactory Pb prediction results under small sample conditions. These findings are consistent with existing research. Specifically, Tan et al. (2021) applied CARS in feature selection and PLS in modeling to predict soil Pb content, achieving a maximum R^2^ of 0.60 in the validation set [37]. Arif et al. (2022) selected feature wavelengths and applied PLS for modeling, achieving an optimal R^2^ of 0.66 [34]. Chen et al. (2022) used fractional-order derivatives for feature selection and combined PLS and SVM regression to predict Pb content, with R^2^ ranging between 0.54 and 0.59, which is insufficient for high-accuracy predictions [7].

In contrast, the transfer learning model ResNet-pH-Pb in this study significantly improved Pb prediction performance in the target domain (R^2^ = 0.77). The R^2^ box plot (Figure 6) shows that the R^2^ distribution of ResNet-pH-Pb is more stable, with improvements of 0.30, 0.34, and 0.47 over SVM-Pb, PLS-Pb, and ResNet-Pb, respectively. Although there remains room for improvement, an R^2^ of 0.77 is notable given the small sample size and high heterogeneity of the Gejiu dataset, surpassing typical accuracies of 0.54–0.66 reported in previous studies [7,34,37]. Transfer learning has been explored in soil spectroscopy to some extent. For instance, Kok et al. (2024) applied transfer learning to improve pH prediction, achieving an R^2^ of 0.66 [15]. However, most of these studies focus on predicting single soil properties, with fewer studies addressing cross-property transfer learning applications. In this study, we innovatively implemented cross-component transfer from pH to Pb, leveraging the high accuracy of the source domain model ResNet-pH (R^2^ = 0.91). By sharing spectral features (e.g., both pH and Pb are governed by soil organic matter and iron oxides), the model reduces overfitting in the small sample target domain and improves its generalization ability.

### 4.2. Feature Analysis of Wavelength Contribution

Wavelength contribution analysis further reveals the predictive mechanism of transfer learning. Here, SHAP values were employed to evaluate wavelength contributions in the source domain model ResNet-pH (Figure 7a) and the transfer learning model ResNet-pH-Pb (Figure 7b). It was found that both models showed high contributions in the 550–750 nm and 1600–1700 nm bands. These bands are associated with the absorption characteristics of soil organic matter, iron oxides (550–750 nm), and water and organic matter (1600–1700 nm) [38], while organic matter and iron oxides are the main adsorbents of Pb [33]. This finding suggests that, although pH and Pb are different components, the spectral features learned by the source domain model can still be effectively transferred to the target domain. This feature sharing originates from the potential correlation between pH and Pb in soil. In the tin mining area, the soil pH is typically acidic, primarily driven by sulfide mineral oxidation generating sulfuric acid [3,39,40]. Low pH values lead to the dissolution of iron oxides, releasing adsorbed Pb, which increases its mobility [41,42]. This acidic environment provides the chemical basis for cross-component transfer, and the spectral features learned by the source domain model ResNet-pH can effectively transfer to the target domain, alleviating overfitting in small sample scenarios and significantly improving Pb prediction accuracy.

Although pH and Pb predictions share some key wavelengths, transfer learning also optimizes the model’s adaptability to the target domain. The contribution of ResNet-pH-Pb significantly increased in the 919 nm, 959 nm, 1639 nm, and 1670 nm bands. The 919 nm and 959 nm bands align with absorption features of clay minerals (O-H stretching) and carbonates (C-O stretching), as documented in soil spectroscopy studies [43,44,45]. These components are critical for Pb prediction, as Pb often forms lead carbonates or is adsorbed onto clay surfaces in contaminated soils [46]. The 1639 nm and 1670 nm bands correspond to water (H-O-H bending) and organic matter (C-H stretching) absorptions, consistent with Pb hydrolysis and organic matter complexation in soils [43,47]. Furthermore, the SHAP contribution to Pb prediction slightly increased in the visible light region (600–700 nm), potentially linked to enhanced spectral interactions of Pb with soil iron oxides and organic matter [33]. Existing studies have also shown that key wavelengths for Pb prediction typically include 600–800 nm, 1390–1460 nm and 1870–1960 nm [48,49]. The 550–750 nm and 1600–1700 nm bands identified here align closely with prior research, confirming their association with Pb adsorption mechanisms. Compared with the SHAP values analyzed for single-component predictions by Zhong et al. (2024), this study innovatively reveals the feature sharing and adaptation mechanisms of cross-component transfer through SHAP value analysis [22].

**Figure 8:**
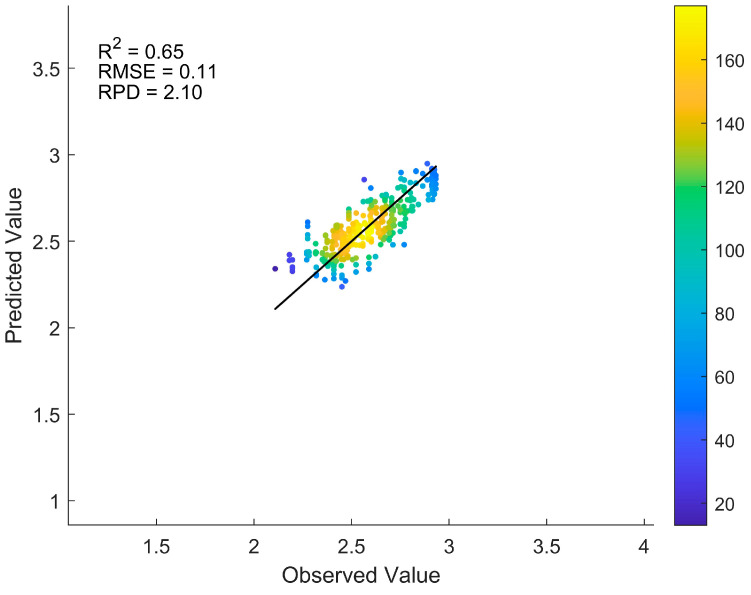
Predicted versus observed Pb values from the transfer learning model (ResNet-pH-Pb) with Box–Cox transformation based on 10 random sampling tests. The average evaluation metrics across tests are shown in the upper-left corner.

To explore alternative preprocessing methods for making the Gejiu Pb dataset (skewness = 3.12, kurtosis = 9.1) more normally distributed, we applied the Box–Cox transformation and compared its performance with the logarithmic transformation used in Section 3.4. The Box–Cox-transformed model (ResNet-pH-Pb) yielded an average R^2^ of 0.65, RMSE of 0.11, and RPD of 2.10, as shown in the scatter plot of predicted versus observed Pb values in Figure 8. Compared to the logarithmic transformation (R^2^ = 0.77, RMSE = 0.59, RPD = 2.12), the Box–Cox transformation yielded lower R^2^ and RPD values, indicating reduced explained variance and prediction reliability. Although the RMSE was significantly lower, it is not directly comparable due to differences in the value ranges after transformation. These findings indicate that the logarithmic transformation is more suitable for this dataset.

### 4.3. Limitations and Future Directions

This study demonstrated the effectiveness of the transfer learning approach in achieving relatively high accuracy in Pb prediction. However, the dataset is limited to 130 samples from a single mining area, which may restrict the model’s ability to generalize to other regions with different soil types and contamination patterns. Future work should incorporate multi-site datasets to improve robustness across diverse soil types. Integrating additional environmental variables, such as soil moisture and proximity to pollution sources, could further enhance prediction accuracy. Additionally, extending the model to predict other heavy metals (e.g., Cd, Zn) and incorporating soil physicochemical properties (e.g., organic matter content) could broaden its utility for environmental monitoring.

## 5. Conclusions

This study successfully achieved cross-component transfer prediction from pH to Pb and, for the first time, utilized SHAP values to analyze wavelength contributions in transfer learning models, innovatively broadening the use of soil spectroscopy for heavy metal assessment. The source domain model ResNet-pH demonstrated high accuracy on the LUCAS dataset (R^2^ = 0.91). In the target domain of the Gejiu mining area, traditional direct modeling methods (SVM-Pb, PLS-Pb, ResNet-Pb) showed low prediction performance (R^2^ < 0.5 for all). The transfer learning model ResNet-pH-Pb significantly improved prediction accuracy (R^2^ = 0.77), with R^2^ values 0.30, 0.34, and 0.47 higher than SVM-Pb, PLS-Pb, and ResNet-Pb, respectively, confirming the advantages of transfer learning in small sample target domains. Wavelength contribution analysis revealed high contributions in the 550–750 nm and 1600–1700 nm bands, further elucidating the feature-sharing mechanism between pH and Pb in highly heterogeneous soils (CV = 2.02, skewness = 3.12). This study demonstrates that transfer learning methods not only markedly enhance the prediction accuracy of small sample target domains but also provide a robust and efficient approach for evaluating soil heavy metal contamination, offering a rapid, non-destructive tool with significant potential for contamination monitoring and environmental management in mining regions.

## Figures and Tables

**Table 1:** ResNet model architecture parameters.

Layer	Type	Filters	Kernel Size	Stride	Width	Number of Parameters	Actication
1	Input	-	-	-	2100	0	-
2	AvgPool	-	10×	1	210	0	-
3	Convolutional	48	3 × 1	1	210	192	Leaky Relu (alpha = 0.01)
4	Maxpooling	-	2 × 1	-	105	0	-
5	Residual Block	48	3 × 1	1	105	6960	Relu
		48	3 × 1	1	105	6960	-
6	Residual Block	48	3 × 1	1	105	6960	Relu
		48	3 × 1	1	105	6960	
7	Convolutional	32	3 × 1	1	105	4640	Leaky Relu (alpha = 0.01)
8	Maxpooling	-	2 × 1	-	52	0	-
9	Flatten	-	-	-	1664	0	-
10	Dense (Fully connected)	-	-	-	16	16,640	Leaky Relu (alpha = 0.01)
11	Dense (Fully connected)	-	-	-	10	170	Leaky Relu (alpha = 0.01)
12	Output	-	-	-	1	11	Relu

**Table 2:** Training and layer freezing details of ResNet models.

Model	Dataset	Training Samples	Testing Samples	Trained Layers	Frozen Layers
ResNet-pH	LUCAS	14,277	4759	All	None
ResNet-Pb	Gejiu	98	32	All	None
ResNet-pH-Pb	Gejiu	98	32	Max-pooling, Dense	Convolutional, Residual

**Table 3:** Summary Statistics of Soil Pb Concentrations (Gejiu) and Soil pH Values (LUCAS).

Dataset	Sample Size	Property	Min	Max	Mean	SD	CV	Skew	Kurt	Median
LUCAS	19,036	pH	3.21	10.08	6.02	1.35	0.22	−0.07	−1.24	6.21
Gejiu	130	Pb	34.6	9720	974.06	1969.17	2.02	3.12	9.1	232

## Data Availability

The original contributions presented in this study are included in the article. Further inquiries can be directed to the corresponding authors.
